# Zn and Se abrogate heavy metal mixture induced ovarian and thyroid oxido-inflammatory effects mediated by activation of NRF2-HMOX-1 in female albino rats

**DOI:** 10.1016/j.crtox.2022.100098

**Published:** 2022-12-22

**Authors:** Boma F. Eddie-Amadi, Anthonet N. Ezejiofor, Chinna N. Orish, Orish E. Orisakwe

**Affiliations:** a World Bank Africa Centre of Excellence in Oilfield Chemicals Research (ACE-CEFOR), University of Port Harcourt, PMB, 5323 Port Harcourt, Choba, Nigeria; b African Centre of Excellence for Public Health and Toxicological Research (ACE-PUTOR), University of Port Harcourt, PMB, 5323 Port Harcourt, Choba, Nigeria; c Department of Anatomy, Faculty of Basic Medical Sciences, College of Health Sciences, University of Port Harcourt, PMB, 5323 Port Harcourt, Choba, Nigeria

**Keywords:** Heavy metal mixture, Essential trace elements, Oxido-inflammatory effects, Ovary, Thyroid gland

## Abstract

The thyroid is vital for the proper functioning of the female reproductive system since it regulates the metabolism and development of ovary. This is an evaluation of the essential trace elements ETE on the heavy metals mixture HMM mediated oxido-inflammatory effects in the ovary and thyroid of female albino rats. Eight groups (5 female rats /group) were treated as follows for 60 days: Group 1: Deionized water only; Group 2: (Pb, Hg, Mn and Al); Group 3: HMM + ZnCl_2_, 0.80 mg/kg; Group 4: HMM + Na_2_SeO_3_, 1.50 mg/kg; Group 5: HMM + ZnCl_2_, 0.80 mg/kg and Na_2_SeO_3_, 1.50 mg/kg combined. On day 60 animals were euthanized, ovary and thyroid were harvested and used for, MDA, NO, antioxidants, TNF-α, IL-6, HMOX-1, Caspase-3, NF-KB, NRF2, HM and histopathology. There was significant bioaccumulation of Pb, Al, Hg and MN; elevated IL-6 and TNF-α, MDA and NO, caspase-3 and NRF2, NFKB and HMOX-1 with significant decrease in antioxidants in the HMM only group in comparison to the control. Co-treatment with ETE reversed most of these effects.

ETE may ameliorate HMM -induced ovarian and thyrotoxicity in female albino rats by blunting oxido-inflammatory activities.

## Highlights

• Ovarian and Thyroidal Pb, Al, Hg and Mn levels after HMM exposure were reduced by Zn and Se.
• Elevated Ovarian and Thyroidal IL-6, TNF, NF-κB, MDA and NO and Cas-3 after HMM exposure were reduced by Zn and Se.
• Decrease in Ovarian and Thyroidal antioxidants and histopathological alterations after HMM exposure were reversed by Zn and Se.

## Graphical abstract

**Figure:**
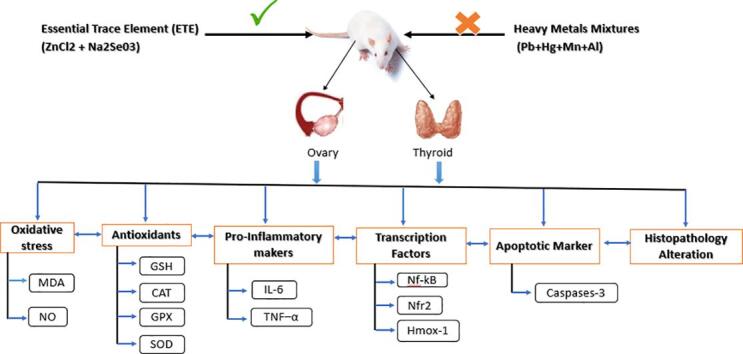

## Introduction

The thyroid is vital for the proper functioning of the female reproductive system, since it regulates the metabolism and development of ovarian, uterine, and placental tissues (Maruo et al., 1992, Silva et al., 2018). Given the physiological and functional relationship between the hypothalamic–pituitary–thyroid and hypothalamic–pituitary–ovarian axes, the thyroid can modulate ovarian functions that may culminate in diminished fecundity (Baldridge et al 2003). It can also act indirectly via multifaceted interactions with other hormones such as estrogen, prolactin (PRL) and by influencing the release of gonadotrophin-releasing hormone (GnRH) in the hypothalamic-pituitary–gonadal axis (Mattheij et al 1995) to influence fertility in both women and animals (Silva et al 2018). Heavy metals are designated as one of the endocrine disrupting chemicals which might have deleterious effects on the reproductive (Ciarrocca et al., 2013, Mendiola et al., 2011). Dietary exposure to these heavy metals (Yang and Massey, 2019) with likelihood of tissue sequestration after exposure have been reported (Mukherjee et al 2022). Earlier investigation uncovered that heavy metals could trigger substantial alterations in the morphology of the uterus and ovary (Wang et al., 2015). Steroidogenesis can be disrupted by presence of heavy metals, which could lead to ovarian necrosis and functional failure (Rafati-Rahimzadeh et al., 2017). Heavy metal associated female infertility demands ameliorative and preventive measures.

Organ toxicity may occur due to exposure to diverse environmental pollutants like heavy metals (Small et al 2012). Oxidative insult is now believed to be a fundamental feature of this predicament that have morphed into a huge public health malady (Zhou et al., 2015, Akchurin and Kaskel, 2015). Oxidative stress is typically under the control of the master cellular sensor the nuclear factor (erythroid-derived 2)-like 2 (Nrf2) which is domiciled in cytosol and strictly regulated by the inhibitory activity of Kelch-like ECH-associated protein 1 (Keap1) (Zhao et al 2019). This transcription factor remains inert until Keap1 is dissociated from it by electrophilic compounds, reactive oxygen species and bioactive nutraceuticals seen in healthy foods. Diverse oxidants and toxic insults like heavy metals distort the segregation to induce the translocation of Nrf2 to the nucleus and its binding to the antioxidant response element (ARE) and expression of antioxidant genes, including NADPH-quinone oxidoreductase 1 (NQO1) and heme oxygenase 1 (hmox-1) (Zhang et al., 2019, Qin et al., 2019, Shi et al., 2019). In the event of cellular stress, hmox-1 is expressed to protect cells from ROS generation (Lee et al 2019). Heme oxygenase 1 (hmox-1) catalyzes the conversion of heme to biliverdin, iron, and carbon monoxide, to scavenge free radicals directly (Shin et al 2019). With progressive chronic oxidative stress, apoptosis (program cell death PCD) sets in to prevent degeneration and proliferation in an uncontrollable way (Linkermann, 2016). Therefore, activation of Nrf2 can alleviate the toxicity related to ROS and is an important molecular target for the prevention of tissue injury (Lu et al 2019).

The thyroid gland which plays an integral role in the maintenance of normal body physiological status is one of the main targets of endocrine disruptors like the heavy metals (Tebourbi et al 2010). The ETE selenium (Se) and zinc (Zn) participate in thyroid function (Mahmoodianfard et al., 2015, Wiersinga, 2016), Salah et al 2022). Se is an indispensable component of selenocysteine- containing selenoproteins like cytoprotective glutathione peroxidases (Schomburg, 2011). On the other hand, Zn is an integral moiety of endogenous enzymatic antioxidant system which ensures cell membrane integrity in diverse cellular (Catania et al., 2009, Formigari et al., 2007) including cytoprotection against free radical damage through maintenance of adequate level of metallothioneins. Zn is also a fundamental component of Cu, Zn-superoxide dismutase (Cu, Zn-SOD) (Formigari et al 2007).

Humans are equipped with enzymatic and non-enzymatic defense mechanisms against either ROS generation or its detoxification (Chen and Kunsch, 2004). Often this in-built versatile defensive machinery may be overwhelmed by the deleterious effects of exaggerated ROS the usual feature of different pathophysiological states (Manna et al., 1997, Saha et al., 2016). It is plausible therefore to earmark the cellular imbalance between antioxidants and pro-oxidants. Understanding the dynamics of oxidative injury which precedes apoptosis (Bhattacharya et al 2011), may be a worthwhile and effectual approach in the management of many pathophysiological conditions. Exogenous supplements with antioxidant activity, which augment the amount and upregulate activities of endogenous antioxidants (Sinha et al., 2015, Rashid and Sil, 2015, Sinha and Sil, 2015) may suffice as credible therapeutic alternatives in combating over generation of ROS (Vitaglione et al., 2005, Sinha et al., 2007, Saha et al., 2016).

The consistent observation of impaired reproductive function by environmental exposure to toxic chemicals, including heavy metals like Pb, Al, Hg, and Mn necessitated this study (Orisakwe, 2021). Crude oil exploration, artisanal refinery and sundry foundry activities in Niger delta, Nigeria have impacted negatively and added to the environmental burden of heavy metals (Pb, Hg, Al and Mn) with attendant public health issues which demand urgent attention and need to harness available remedies (Okoye et al., 2021, Liu et al., 2018, Su et al., 2016). Furthermore, some antioxidants are known to exert anti-apoptotic effect in heavy metal-induced damage via inhibition of oxidative stress due to activated Nrf2 signalling (Deng et al., 2015). We hypothesized that ETE inhibit HMM -induced oxidative stress, inflammatory response, and apoptosis by activating the NRF2/HMOX-1 pathway and explored the mechanism by which ETE may impede ovarian and thyroid injury in HMM exposed female albino rats.

## Materials and methods

### Chemicals

Heavy metals such as Lead acetate, Alumnium chloride, Mercury Chloride and Manganese dichoride were purchased from Sigma Chemical Co. (St. Louis, MO, USA). Tumor necrosis factor alpha (TNF – α), interleukin 6 (IL – 6), Caspase-3, Nuclear factor erythriod 2- related factor 2 (NRF2), Nuclear factor kappa B (NF-KB) and Heme Oxygynase – 1 (HMOX-1) Rat ELISA Kit were purchased from Elab Science Biotechnology Company, (Beijing, China). All other reagents were of analytical grade and were obtained from the British Drug Houses (Poole, Dorset, UK).

### Animals and treatments

A total number of 25 healthy female Sprague Dawley rats aged between 6 and 8 weeks were purchased from the Department of Pharmacology, Animal House, University of Port Harcourt, Rivers State. Animals were housed in standard polypropylene cages, that do not release endocrine disruptors under room temperature 25 ± 2 °C with a 12-h light/dark cycles throughout the duration of the experiment (Kang et al 2011). Prior to the commencement of the study, the animals were acclimatized for two weeks. Standardized protocol that employed the ARRIVE guidelines (Animal Research: Reporting In Vivo Experiments) checklist (Du Sert, et al 2020) was used, and ethical approval was obtained from the University of Port Harcourt institutional Centre for Research Management and Development Animal Care and Use Research Ethics Committee (UPH/CEREMAD/REC/18). The experiment was conducted in accordance with the “Guide for the Care of Laboratory Animals” approved by the National Academy of Science (NAS). The animals received standard feed and deionized water *ad libitum*.

### Experimental design

The experimental animals were weight matched and divided into 5 groups of five female Sprague Dawley rats each. Treatments were by oral gavage to precisely administer a fixed volume. The doses of the heavy metal mixture (Pb, (20mgkg^−1^), Hg (0.40mgkg^−1^), Mn (0.560mgkg^−1^) and Al (35mgkg^−1^)) (Eddie-Amadi et al 2022) reflective of concentrations recorded in various environmental matrices in the Niger delta, Nigeria (Okoye et al 2021). The animals’ treatment protocol detailed as follows Table 1 lasted for 60 days:

**Table 1:** Experimental design.

S/**N**	**Experimental group**	**Treatment**
1	Group 1 (Control)	Deionized water only
2	Group 2	heavy metals mixture HMM only Pb, (20mgkg^−1^), Hg (0.40mgkg^−1^), Mn (0.560mgkg^−1^) and Al (35mgkg^−1^).
3 4 5	Group 3 Group 4: Group 5	received HMM + ZnCl_2_, 0.80 mg/kg (Anyanwu et al 2020) HMM + Na_2_SeO_3_, 1.50 mg/kg (Messarah et al 2012) HMM + ZnCl_2_, 0.80 mg/kg and Na_2_SeO_3_, 1.50 mg/kg combined

### Body, ovary and thyroid weight, feed and fluid consumption

The body weight of rats was measured weekly and at the end of the experiment on the 60th day, while the ovary and thyroid weight was recorded directly after the sacrifice on the 60th day.

The absolute weight of the ovary and thyroid (g) = mean of ovary and thyroid weight for each group were taken. The relative weight of ovary and thyroid (g/100 g body weight) = mean of ovary and thyroid weight for each group/final body weight × 100 were calculated. Feed (g) and fluid consumption (ml) were recorded daily.

### Necropsy and harvesting of ovary and thyroid

After 60 days of treatment, animals in each group were euthanized under pentobarbital (50 mg/kg IP) anaesthesia. The ovary and thyroid of each rat were harvested, rinsed in cold saline water, weighed, and used for both biochemical parameters and heavy metal analyses.

### Metal analysis

Twenty milligrams of ovary and thyroid were digested separately using 2 ml of perchloric acid and 6 ml of nitric acid. Afterwards, the samples were kept for 30 min before heating at 105 ◦C until digestion was completed. The solution was made up to 15 ml (final volume) with deionized water. Solar thermo elemental flame Atomic Absorption Spectrometer (Model SG 71906) was used to determine the Lead (Pb), Alumnium (Al), Mercury (Hg) and Manganese (Mn) concentrations Okoye et al 2021. The limits of detection (LoD) were 0.001 mg/Kg for Alumnium (Al), Mercury (Hg) and Manganese (Mn) and 0.01 mg/Kg for Pb, while the limits of quantification (LoQ) were 0.0033 mg/Kg for Alumnium (Al), Mercury (Hg) and Manganese (Mn) and 0.033 mg/Kg for Pb.

### Determination of hormonal profile markers.

The concentrations of follicle stimulating hormone (FSH), luteinizing hormone (LH), prolactin (PRL) and progesterone (PROG) were assayed using the enzyme –linked immunosorbent assay kits (ELISA microwells, monobind Inc. Lake Forest, CA 92630, USA).

### Assessment of oxidative stress markers

The malondialdehyde MDA level (lipid peroxidation marker) was assayed using the procedure of Ohkawa and Ohishi (1979). In this technique, MDA reacts with the chromogenic reagent, 2-thiobarbituric acid (TBA) under acidic medium to produce a pink coloured complex at 532 nm absorbance.

Nitric oxide (NO) level was assayed according to the method of Green et al., (1982).

### Assay of antioxidants

Briefly, glutathione peroxidase (GPx) activity was assayed according to the method of Rotruck et al. (1973). Reduced glutathione (GSH) levels were assessed using the technique illustrated by Jollow et al. (1974). Catalase (CAT) activity was evaluated using the technique of Clairborne (1995) with slight modification. This technique is premised on the principle that catalase in the sample will split hydrogen peroxide which can be estimated at 240 nm using a spectrophotometer (Clairborne, 1995). Superoxide dismutase (SOD) activity was estimated with the technique previously illustrated by Misra and Fridovich (1972). This technique is based on the principle that at pH 10.2, SOD has the capacity to inhibit the autoxidation of epinephrine.

### Enzyme-linked immunosorbent assay

The content of the inflammatory cytokines including tumor necrosis factor-α (TNF-α), interleukin-6 (IL-6), heme oxygenase and Hmox-1, apoptotic marker caspase 3, and transcription factors NF-KB, NRF2 in the homogenized thyroid cell supernatant was detected using commercially available ELISA kits following the manufacturer’s instructions. All experiments were conducted in triplicate.

Assessment of inflammatory markers and transcription factor markers

IL-6, TNF-α, NRF2, HMOX-1, NFKB and Caspase 3 activities were measured with IL-6, TNF-α, NRF2, HMOX-1, NFKB and Caspase 3 Activity Assay Kit (Beyotime Institute of Biotechnology, Jiangsu, China) according to the manufacturer's directions. Ovary and thyroid samples were lysed for 15 min on ice. The ovarian and thyroid homogenates were centrifuged separately at 16,000g for 10 min at 4 °C.

Briefly, standards or samples were added to the micro-ELISA plate wells and combined with the specific antibody. Then a biotinylated detection antibody specific for rat IL-6 and Avidin-Horseradish Peroxidase (HRP) conjugate were added successively to each micro plate well and incubated. Free components were washed away. The substrate solution was added to each well. Only those wells that contain rat IL-6, biotinylated detection antibody and Avidin-HRP conjugate appeared blue in color. The enzyme-substrate reaction was terminated by the addition of stop solution and the color turned yellow. The optical density (OD) was measured spectrophotometrically at a wavelength of 450 nm ± 2 nm. The OD value was proportional to the concentration of rat IL-6. Concentrations of rat IL-6 in the samples were calculated by comparing the OD of the samples to the standard curve. This procedure was repeated for TNF-α, NRF2, HMOX-1, NFKB and Caspase 3. Nitric oxide (NO) level was assayed according to the method of Green et al. (1982).

### Histopathologic examinations

After 10 % neutral buffered formalin fixed 72 h, ovary and thyroid were separately embedded in paraffin by standard histological method., the tissues were sectioned coronally in 5 μm thickness, and then slices were conducted after dewaxing and hydration procedures. The sections were thereafter routinely stained with Hematoxylin-Eosin Stain kit (Vector laboratories, USA) according to the manufacturer's instructions. The sections of the ovary and thyroid were evaluated under a light microscope and photographed with image acquisition parameters settings at 100 × and 400 × throughout the process.

### Statistical analysis

All the results were expressed as Mean ± Standard deviation (std). Microsoft Xlstat 2014 was used in performing Analysis of Variance and Tukey multiple comparison pairwise tests to check if the concentration of the biomarkers was significantly (at 5 % significant level) different between groups. Pandas was used in obtaining the descriptive statistical parameters (biomarkers and heavy metals mean conc.). Seaborn and Matplotlib were used in plotting all graphs. The data analysis involved performing descriptive statistics (mean and std) on the heavy metals and biomarkers concentration before ANOVA was used to establish if there was significant difference in the concentration of the heavy metals and biomarkers among groups. Pearson R correlation was used to understand the relationship among biomarkers.

## Results

### Heavy metal accumulation in the ovary and thyroid of rats treated with ETE following HMM exposure.

**Fig. 1:**
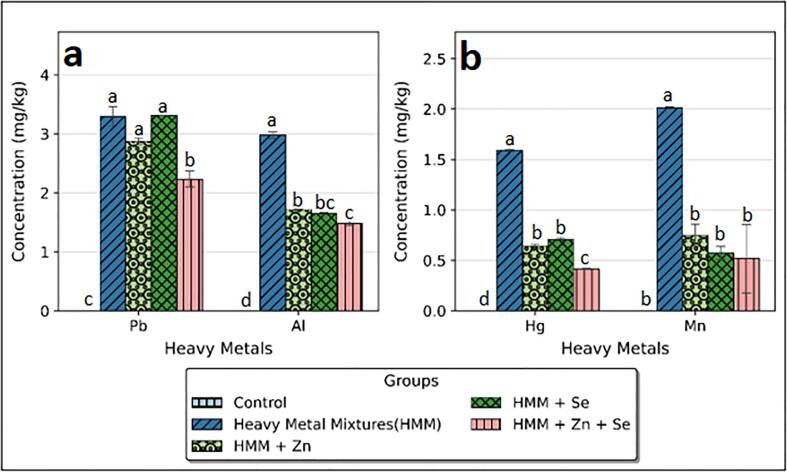
Effect of essential trace elements on the concentrations of Hg, Mn, Pb and Al in the ovary of heavy metal mixture exposed female albino rats. Values with different superscripts (a, b, c) were significantly different from each other (p < 0.05) and those with the same superscripts were not significantly different.

**Fig. 2:**
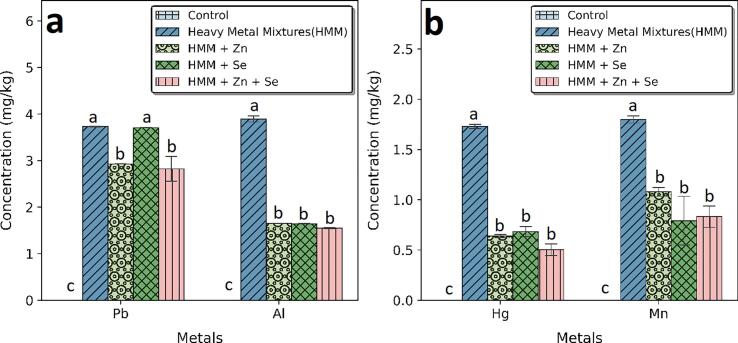
Effect of essential trace elements on the concentrations of Hg, Mn, Pb and Al in the Thyroid of heavy metal mixture exposed female albino rats. Values with different superscripts (a, b, c) were significantly different from each other (p < 0.05) and those with the same superscripts were not significantly different.

The effects of ETE on the concentrations of Pb, Hg. Al, and Mn in the ovary and thyroid of HMM exposed female albino rats are shown in Fig. 1, Fig. 2 respectively. There was marked bioaccumulation of Pb, Hg. Al, and Mn in the ovary and thyroid after HMM exposure.

Zn, Se and Zn + Se combination significantly reduced the Pb, Hg, Al and Mn levels in the ovary. The percentage reductions of lead in the ovary shown by Zn, Se and Zn + Se combination were 13.28, 0.04 and 32.60 % respectively. The percentage reductions of Hg in the ovary shown by Zn, Se and Zn + Se combination were 59.81, 55.51 and 73.94 % respectively. The percentage reductions of Al in the ovary shown by Zn, Se and Zn + Se combination were 42.44, 44.79 and 50.25 % respectively. The percentage reductions of Mn in the ovary shown by Zn, Se and Zn + Se combination were 62.88, 71.59 and 74.15 % respectively Fig. 1 b.

Zn, Se and Zn + Se combination significantly reduced the Pb, Hg. Al, and Mn levels in the thyroid. The percentage reductions of Pb and Al in the thyroid shown by Zn, Se and Zn + Se combination were 21.57, 0.66 and 24.37 %, 57.63, 57.79 and 60.25 % respectively (Fig. 2a). The percentage reductions of Hg and Mn in the thyroid shown by Zn, Se and Zn + Se combination were 63.08, 60.08 and 70.91 %, 40.06, 55.96 and 53.74 % respectively (Fig. 2b).

### Oxidative stress and lipid peroxidation markers of rats treated with ETE following HMM exposure

**Fig. 3:**
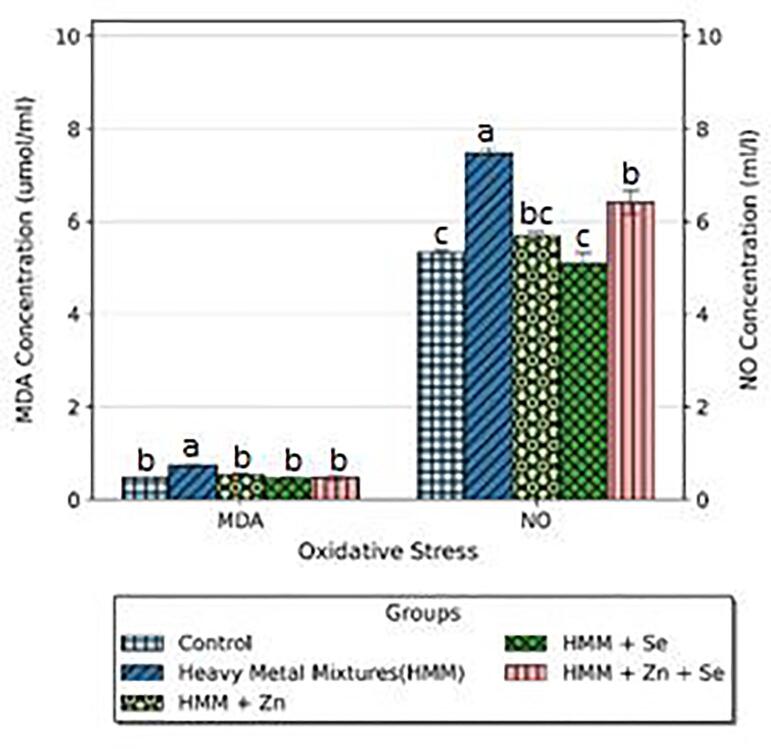
Effect of essential trace elements on the MDA and NO in the ovary of heavy metal mixture exposed female albino rats. Values with different superscripts (a, b, c) were significantly different from each other (p < 0.05) and those with the same superscripts were not significantly different.

The effects of Zn, Se and Zn + Se on the ovarian and thyroidal oxidative stress markers (MDA and NO) of HMM exposed female rats are shown in Fig. 3. There was a significant increase in MDA levels of HMM only exposed rats when compared to the control rats. The NO levels in the HMM-exposed rats are significantly higher than in the control group. The Zn, Se and Zn + Se had a significant decrease in the HMM mediated MDA and NO elevations.

**Fig. 4:**
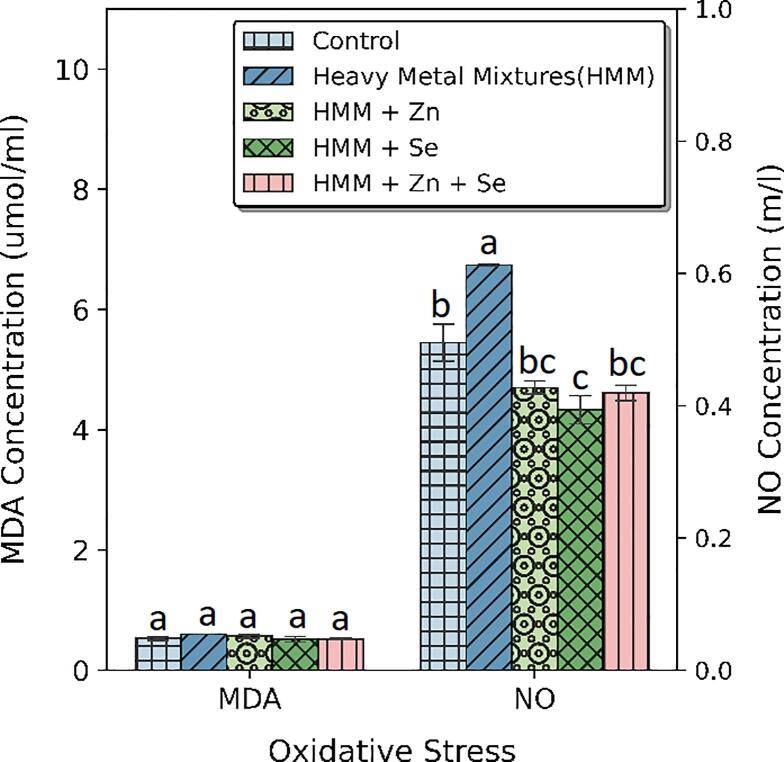
Effect of essential trace elements on the MDA and NO in the Thyroid of heavy metal mixture exposed female albino rats. Values with different superscripts (a, b, c) were significantly different from each other (p < 0.05) and those with the same superscripts were not significantly different.

The effects of Zn, Se and Zn + Se on the oxidative stress markers (MDA and NO) in the thyroid of heavy metal mixture HMM exposed female rats is shown in Fig. 4. The Zn, Se and Zn + Se had a non-significant decrease in the HMM mediated MDA elevation but a significant decrease in in the HMM mediated NO elevation (Fig. 4).

### Antioxidants profile of rats treated with ETE following HMM exposure.

**Fig. 5:**
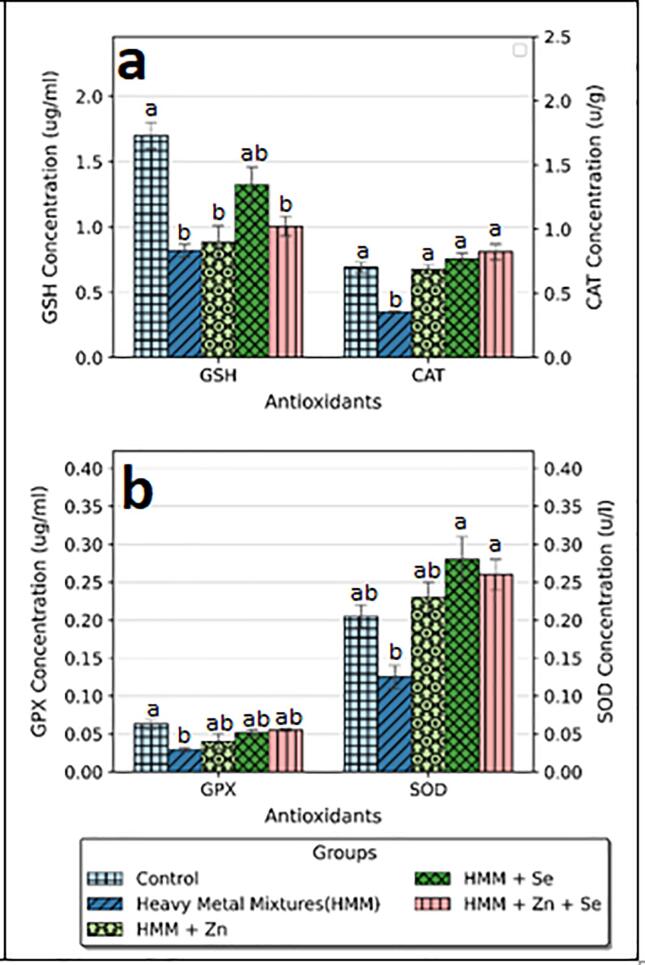
**E**ffect of essential trace elements on the antioxidant profile in the ovary of heavy metal mixture exposed female albino rats. Values with different superscripts (a, b, c) were significantly different from each other (p < 0.05) and those with the same superscripts were not significantly different.

The effects of Zn, Se and Zn + Se on the ovarian antioxidant markers (SOD, CAT, GSH and GPx) of HMM exposed female rats are shown in Fig. 5. HMM only exposed rats caused significant decrease the antioxidant markers (SOD, CAT, GSH and GPx) when compared with control (Fig. 5a). The ETE (Zn, Se and Zn + Se) had significant increase on the antioxidant markers (SOD, CAT, GSH and GPx) in comparison to the HMM only exposed rats (Fig. 5b).

**Fig. 6:**
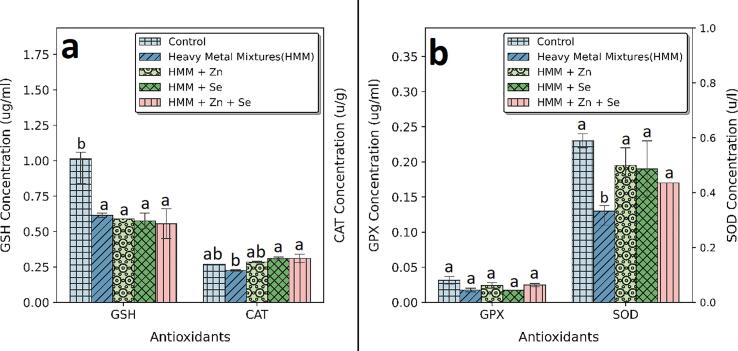
Effect of essential trace elements on the antioxidant profile in the Thyroid of heavy metal mixture exposed female albino rats. Values with different superscripts (a, b, c) were significantly different from each other (p < 0.05) and those with the same superscripts were not significantly different.

The effects of Zn, Se and Zn + Se on the thyroidal antioxidant markers (SOD, CAT, GSH and GPx) of HMM exposed female rats is shown in Fig. 6. There was a significant increase in SOD levels following ETE (Zn, Se and Zn + Se) treatments when compared with the HMM only group. The ETE (Zn, Se and Zn + Se) had a non-significant increase in GPx in comparison to the HMM only exposed rats. But the ETE (Zn, Se and Zn + Se) had a significant increase in CAT in comparison to the HMM only exposed rats (Fig. 6a).

The ETE (Zn, Se and Zn + Se) had a significant increase in SOD in comparison to the HMM only exposed rats (Fig. 6b).

### Apoptotic marker (Caspase 3) activities of rats treated with ETE following HMM exposure

**Fig. 7:**
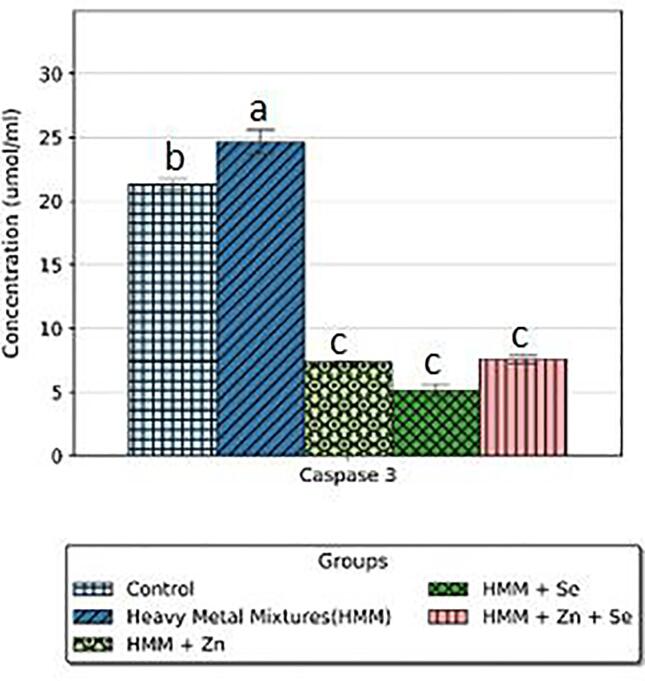
Effect of essential trace elements on the Caspase-3 in the ovary of heavy metal mixture exposed female albino rats. Values with different superscripts (a, b, c) were significantly different from each other (p < 0.05) and those with the same superscripts were not significantly different.

The effects of ETE (Zn, Se and Zn + Se) on the ovarian caspase-3 of HMM exposed female rats are shown in Fig. 7. HMM only exposed rats caused significant increase in the ovarian caspase-3 when compared with control. The (Zn, Se and Zn + Se) had significant decrease on the ovarian caspase-3 in comparison to the HMM only exposed rats.

**Fig. 8:**
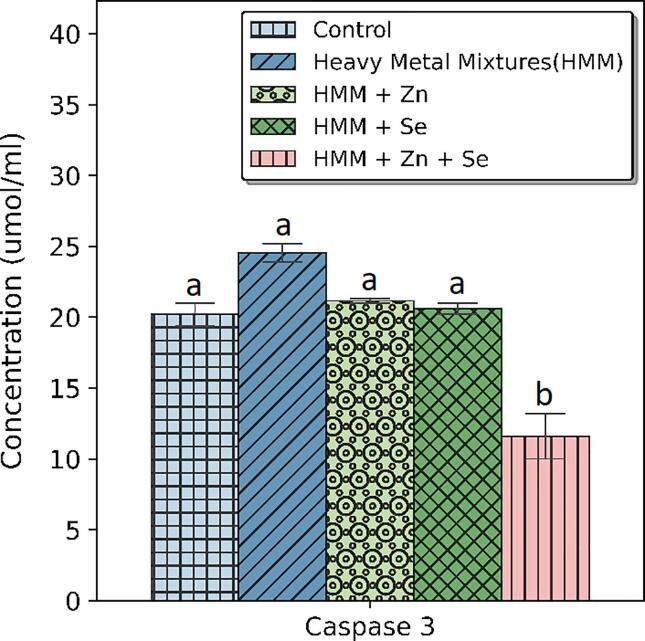
Effect of essential trace elements on the Caspase-3 in the Thyroid of heavy metal mixture exposed female albino rats. Values with different superscripts (a, b, c) were significantly different from each other (p < 0.05) and those with the same superscripts were not significantly different.

The effects of ETE (Zn, Se and Zn + Se) on the caspase-3 (thyroid) of HMM exposed female rats is shown in Fig. 8. HMM caused non-significant increase on the caspase-3 (thyroid) when compared with control. The ETE (Zn, Se) had non-significant decrease on the caspase-3 (thyroid) in comparison to the HMM only exposed rats. Zn + Se supplementation had significant reduction of the caspase-3 (thyroid) in comparison to HMM only exposed rats (Fig. 8).

### Pro-Inflammatory markers of rats treated with ETE following HMM exposure

**Fig. 9:**
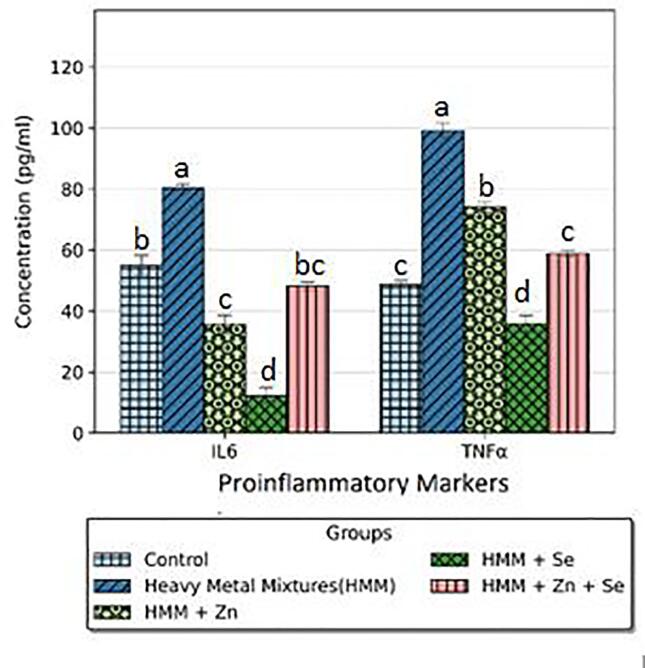
Effect of essential trace elements on the Pro-Inflammatory markers Interlukin-6 (IL-6) and Tumor necrosis factor alpha (TNF – α) in the ovary of heavy metal mixture exposed female albino rats. Values with different superscripts (a, b, c) were significantly different from each other (p < 0.05) and those with the same superscripts were not significantly different.

The effects of ETE (Zn, Se and Zn + Se) on the ovarian pro-inflammatory markers (IL-6 and TNF-α) of HMM exposed female rats are shown in Fig. 9. HMM treatment resulted in a significant increase in TNF-α and IL − 6 levels in the ovary when compared with the control. The (Zn, Se and Zn + Se) had significant decrease on the ovarian pro-inflammatory markers (IL-6 and TNF-α) in comparison to the HMM only exposed rats. Selenium alone had more significant reduction of the ovarian pro-inflammatory markers (IL-6 and TNF-α) in comparison to the Zn and Zn + Se exposed rats treated rats.

**Fig. 10:**
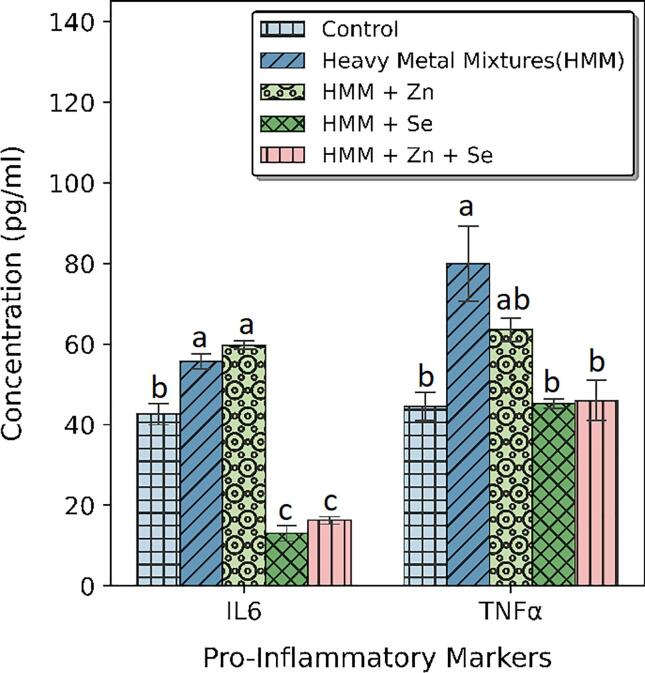
Effect of essential trace elements on the Pro-Inflammatory markers Interlukin-6 (IL-6) and Tumor necrosis factor alpha (TNF – α) in the Thyroid of heavy metal mixture exposed female albino rats. Values with different superscripts (a, b, c) were significantly different from each other (p < 0.05) and those with the same superscripts were not significantly different.

The effects of ETE (Zn, Se and Zn + Se) on the thyroidal pro-inflammatory markers (IL-6 and TNF-α) of HMM exposed female rats is shown in Fig. 10. HMM exposure resulted in a significant increase in TNF-α and IL − 6 levels in the thyroid when compared with the control. The ETE (Zn, Se and Zn + Se) had significant decrease on the thyroidal pro-inflammatory markers (IL-6 and TNF-α) in comparison to the HMM only exposed rats (Fig. 10).

### Transcription factors (NF-ΚB and NRF2) and, HMOX-1 of rats treated with ETE following HMM exposure in the ovary and thyroid.

**Fig. 11:**
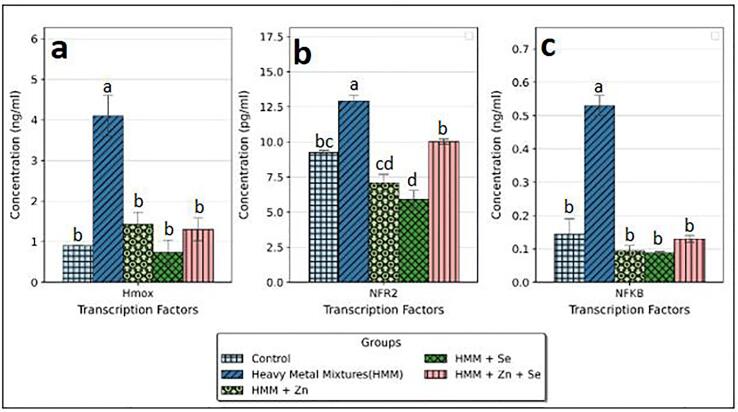
Effect of essential trace elements on the Transcription factors (NfkB, and Nfr2) and Hmox-1 in the ovary of heavy metal mixture exposed female albino rats. Values with different superscripts (a, b, c) were significantly different from each other (p < 0.05) and those with the same superscripts were not significantly different.

The effects of ETE (Zn, Se and Zn + Se) on the ovarian transcription factors (NF-KB and HMOX-1) of HMM exposed female rats is shown in Fig. 11. There were significantly increased ovarian levels of NF-ΚB, HMOX-1 and NRF2 following HMM exposure when compared with the control. The ETE (Zn, Se and Zn + Se) had significant decrease in the ovarian transcription factors (NF-KB (Fig. 11c) and Hmox-1 (Fig. 11a) in comparison to the HMM only exposed rats. Selenium alone had more significant reduction of the ovarian HMOX-1 in comparison to the Zn and Zn + Se exposed rats treated rats.

**Fig. 12:**
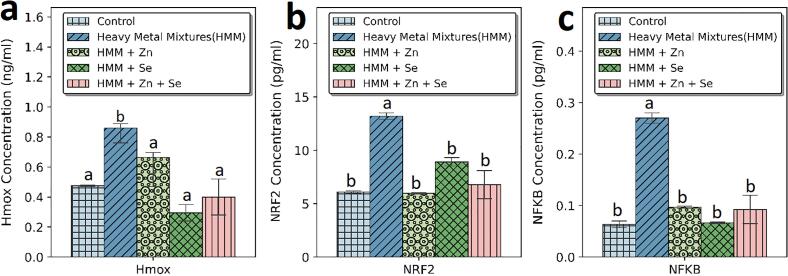
Effect of essential trace elements on the Transcription factors (NfkB, and Nrf2) and Hmox-1 in the Thyroid of heavy metal mixture exposed female albino rats. Values with different superscripts (a, b, c) were significantly different from each other (p < 0.05) and those with the same superscripts were not significantly different.

The effects of ETE (Zn, Se and Zn + Se) on the thyroidal transcription factors (NF-KB, and NRF2) and Hmox-1 of HMM exposed female rats is shown in Fig. 12. HMM only exposure caused increase in NRF2 level (Fig. 12b). This increase was significant in comparison to control. The ETE (Zn, Se and Zn + Se) had significant decrease on the thyroidal transcription factors (NF-KB (Fig. 12c) and Hmox-1 (Fig. 12a)) in comparison to the HMM only exposed rats. Selenium alone had more significant reduction of the thyroidal HMOX-1 in comparison to the Zn and Zn + Se exposed rats treated rats. Zn showed higher level of HMOX-1 than control group (Fig. 12a). Se showed higher level of NRF2 than control ((Fig. 12b).

### Hormonal profile in the ovary of rats treated with ETE following HMM exposure.

**Fig. 13:**
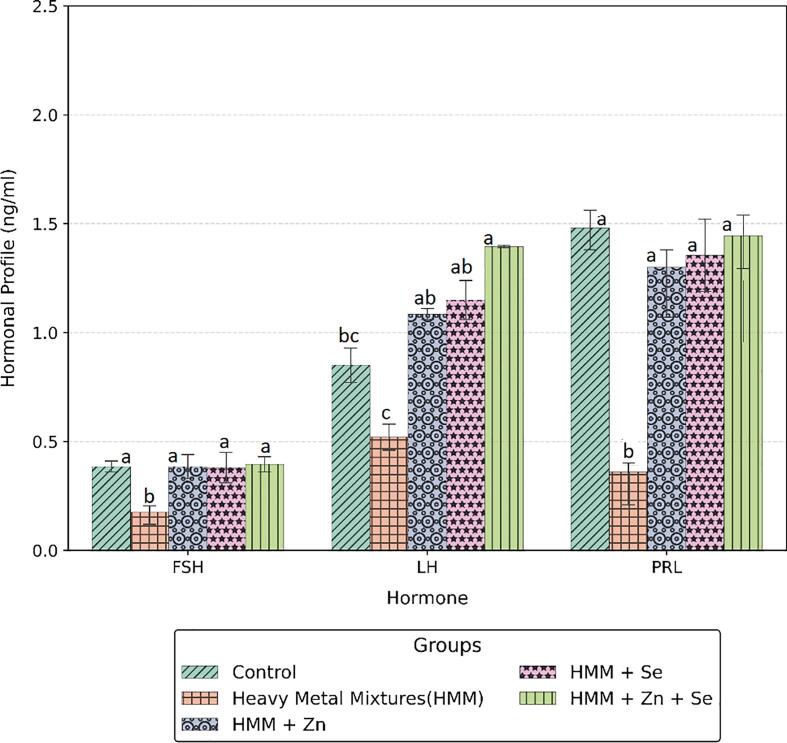
Effects of essential trace elements on hormonal profile (FSH, LH & PRL) of heavy metal mixture exposed female albino rats. Values with different superscripts (a, b, c) were significantly different from each other (p < 0.05) and those with the same superscripts were not significantly different.

**Fig. 14:**
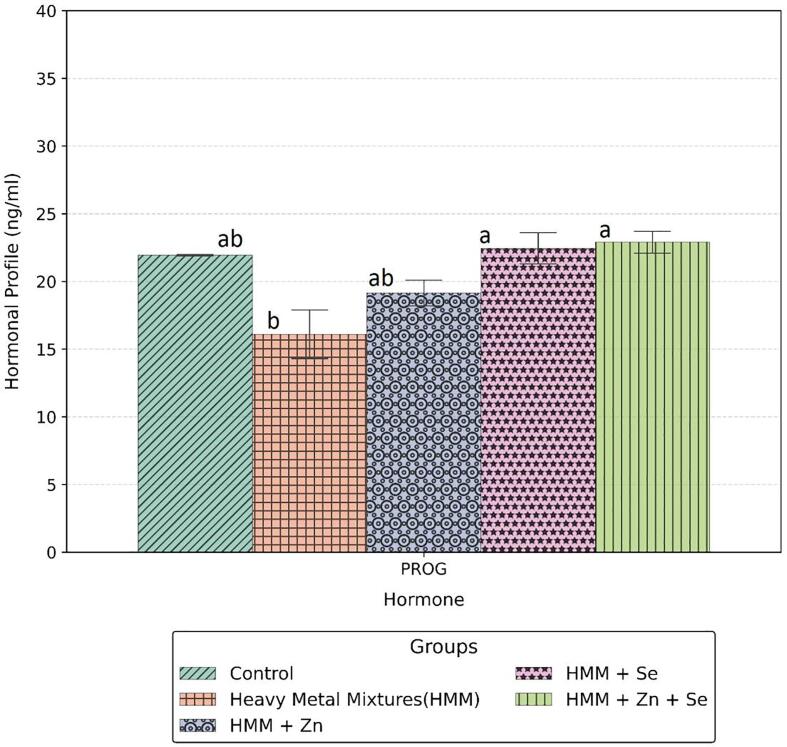
Effects of essential trace elements on hormonal profile (Progesterone) of heavy metal mixture exposed female albino rats. Values with different superscripts (a, b, c) were significantly different from each other (p < 0.05) and those with the same superscripts were not significantly different.

The effects of ETE (Zn, Se and Zn + Se) on the ovarian hormones (FSH, LH, PRL) of HMM exposed female rats are shown in Fig. 13, whereas the effects of ETE (Zn, Se and Zn + Se) on the ovarian progesterone of HMM exposed female rats are shown in Fig. 14. The concentrations of LH, FSH and PROG in the HMM only exposed groups were significantly decreased when compared with the control and ETE (Zn, Se and Zn + Se) treated groups. HMM only exposed rats caused significant decrease in the ovarian progesterone when compared with control. The ETE (Zn, Se and Zn + Se) had significant increase on the ovarian progesterone in comparison to the HMM only exposed rats.

### Effect of ETE on body weight, absolute and relative weight of ovary and thyroid in HMM.

Table 2, Table 3 show the effect of ETE on body weight, absolute and relative weight of ovary and thyroid in HMM treated rats respectively. There was significant difference in the absolute and relative weight of the ovary between the control and HMM only exposed group, and between the HMM only exposed group HMM plus ETE treated groups Table 2. There were no significant differences in the absolute and relatives weights of the thyroid between control and HMM exposed groups, and between HMM only exposed group and the ETE treated groups Table 3.

**Table 2:** Effect of essential trace elements on body weight, absolute and relative weight of Ovaries in heavy metal mixture treated rats.

**GROUP**	**ABSOLUTE ORGAN WEIGHT (g)**	**BODY WEIGHT (g)**	**RELATIVE ORGAN WEIGHT (%)**	**Feed intake (g)**	**Fluid intake (ml)**
Control	0.15 ± 0.04	I = 125.82 ± 16.43 F = 200.00 ± 14.14	0.08 ± 0.04	154.10 ± 12.39	142.98 ± 35.36
HMM	0.25 ± 0.04	I = 116.86 ± 8.05 F = 180.50 ± 0.71	0.14 ± 0.04	148.27 ± 6.52	110.07 ± 33.46
HMM + Zn	0.09 ± 0.01	I = 59.59 ± 4.99 F = 162.50 ± 12.02	0.06 ± 0.01	149.94 ± 11.25	95.62 ± 43.20
HMM + Se	0.08 ± 0.02	I = 75.71 ± 6.99 F = 155.50 ± 0.71	0.05 ± 0.02	127.63 ± 22.17	109.74 ± 51.27
HMM + Zn + Se	0.11 ± 0.02	I = 39.57 ± 6.92 F = 155.00 ± 7.07	0.07 ± 0.02	126.64 ± 24.82	103.85 ± 53.26

Data are expressed as (Mean ± SD).

**Table 3:** Effect of essential trace elements on body weight, absolute and relative weight of Thyroid in heavy metal mixture treated rats.

**GROUP**	**ABSOLUTE ORGAN WEIGHT (g)**	**BODY WEIGHT (g)**	**RELATIVE BODY WEIGHT (%)**	**Feed intake (g)**	**Fluid intake (ml)**
Control	0.50 ± 0.14	I = 125.82 ± 16.43 F = 200.00 ± 14.14	0.25 ± 0.14	154.10 ± 12.39	142.98 ± 35.36
HMM	0.55 ± 0.11	I = 116.86 ± 8.05 F = 180.50 ± 0.71	0.31 ± 0.11	148.27 ± 6.52	110.07 ± 33.46
HMM + Zn	0.47 ± 0.01	I = 59.59 ± 4.99 F = 162.50 ± 12.02	0.29 ± 0.01	149.94 ± 11.25	95.62 ± 43.20
HMM + Se	0.49 ± 0.03	I = 75.71 ± 6.99 F = 155.50 ± 0.71	0.32 ± 0.03	127.63 ± 22.17	109.74 ± 51.27
HMM + Zn + Se	0.46 ± 0.01	I = 39.57 ± 6.92 F = 155.00 ± 7.07	0.3 ± 0.01	126.64 ± 24.82	103.85 ± 53.26

Data are expressed as (Mean ± SD).

### Relationship between biomarkers using Pearson R correlation

In other to establish the relationship between the biomarkers, oxidative stress, pro-inflammatory markers, apoptotic marker and transcription factor Pearson R correlation was used Table 4. Table 4 showed that as the oxidative stress markers (MDA, NO) increases there was a significant reduction in the antioxidants (SOD, CAT, GSH, and GPx). There was a significant negative relationship between oxidative stress markers and antioxidants. The oxidative stress markers had a significant positive relationship with the pro-inflammatory markers (IL6 and TNF-α). An increase in the oxidative stress marker leads to an increase in the pro-inflammatory markers and vice versa. Also, the relationship between the oxidative stress and the NF-KB showed that there was a positive relationship between the two variables. An increase in oxidative stress will result in an increase in the NF-KB and vice versa. Similarly, the result shows positive relationship between oxidative stress and apoptotic marker (Caspase-3).

**Table 4:** Relationship between oxidative stress, antioxidants, pro-Inflammatory marker, apoptotic marker and transcription factors in the thyroid.

Variables	GSH	GPX	CAT	SOD	MDA	Casp 3	Hmox	IL6	NFR2	NFKB	TNFα	NO
GSH	**1.00**											
GPX	0.48	**1.00**										
CAT	0.35	**0.69**	**1.00**									
SOD	**0.66**	0.45	0.08	**1.00**								
MDA	**−0.72**	−0.22	−0.17	**−0.81**	**1.00**							
Casp 3	−0.36	−0.47	−0.50	**−0.68**	**0.72**	**1.00**						
Hmox	−0.38	−0.35	0.05	−0.59	0.48	0.46	**1.00**					
IL6	**−0.71**	−0.46	−0.12	**−0.69**	**0.65**	0.48	0.55	**1.00**				
NFR2	**−0.70**	**−0.75**	−0.41	**−0.72**	0.55	0.62	0.57	**0.91**	**1.00**			
NFKB	**−0.76**	**−0.64**	−0.28	**−0.89**	**0.78**	**0.69**	**0.78**	**0.82**	**0.88**	**1.00**		
TNFα	−0.56	−0.54	−0.45	−0.63	**0.70**	**0.76**	0.24	**0.81**	**0.81**	**0.69**	**1.00**	
NO	**−0.67**	−0.53	−0.54	−0.61	**0.71**	**0.74**	0.25	**0.79**	**0.82**	**0.70**	**0.95**	**1.00**

Values in bold are different from 0 with a significance level alpha = 0.05.

### Histology of the ovary of rats treated with ETE following HMM exposure

**Fig. 15:**
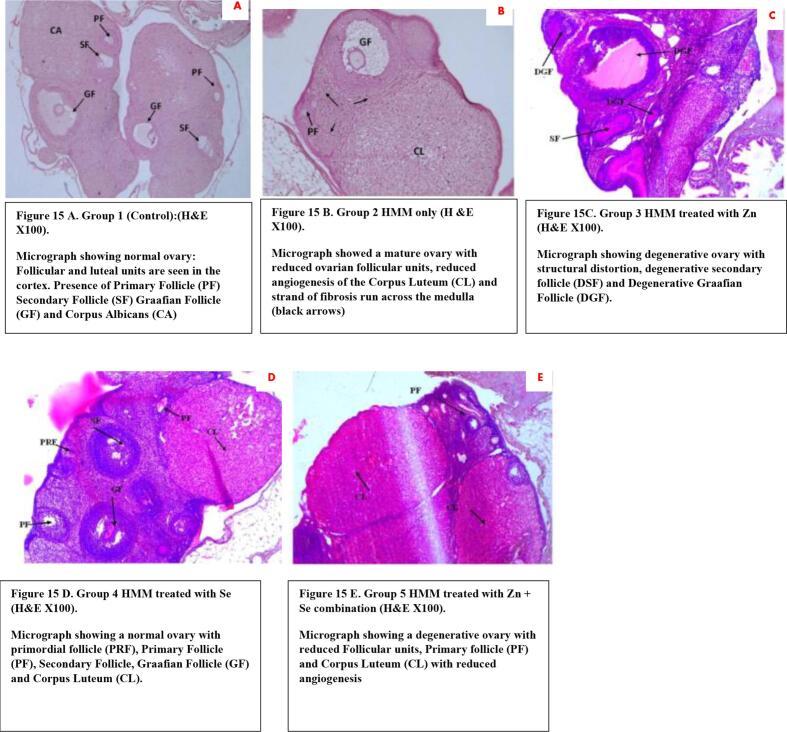
A. Group 1 (Control):(H&E X100). Micrograph showing normal ovary: Follicular and luteal units are seen in the cortex. Presence of Primary Follicle (PF) Secondary Follicle (SF) Graafian Follicle (GF) and Corpus Albicans (CA). Fig. 15 B. Group 2 HMM only (H &E X100). Micrograph showed a mature ovary with reduced ovarian follicular units, reduced angiogenesis of the Corpus Luteum (CL) and strand of fibrosis run across the medulla (black arrows). Fig. 15C. Group 3 HMM treated with Zn (H&E X100). Micrograph showing degenerative ovary with structural distortion, degenerative secondary follicle (DSF) and Degenerative Graafian Follicle (DGF). Fig. 15 D. Group 4 HMM treated with Se (H&E X100). Micrograph showing a normal ovary with primordial follicle (PRF), Primary Follicle (PF), Secondary Follicle, Graafian Follicle (GF) and Corpus Luteum (CL). Fig. 15 E. Group 5 HMM treated with Zn + Se combination (H&E X100). Micrograph showing a degenerative ovary with reduced Follicular units, Primary follicle (PF) and Corpus Luteum (CL) with reduced angiogenesis

There was normal ovarian cortex with primordial follicles, growing follicles (GF) and mature ovum (MO) as well as secondary follicles at different stages of development (Fig. 15 A) while the HMM treated group showed degenerated ovarian cortex (DOC) with follicles and theca cells (Fig. 15B). The ovaries of the Zn treated HMM exposed female rats showed degenerative ovary with structural distortion, degenerative secondary follicle (DSF) and Degenerative Graafia Follicle (DGF), the ovaries of the Se treated HMM exposed female rats showed normal ovary with primordial follicle (PRF), Primary Follicle (PF), Secondary Follicle, Graafian Follicle (GF) and Corpus Luteum (CL). The ovaries of the Zn + Se combination treated HMM exposed female rats showed degenerative ovaries with reduced Follicular units, Primary follicle (PF) and Corpus Luteum (CL) with reduced angiogenesis.

### Histology of the thyroid of rats treated with ETE following HMM exposure

**Fig. 16:**
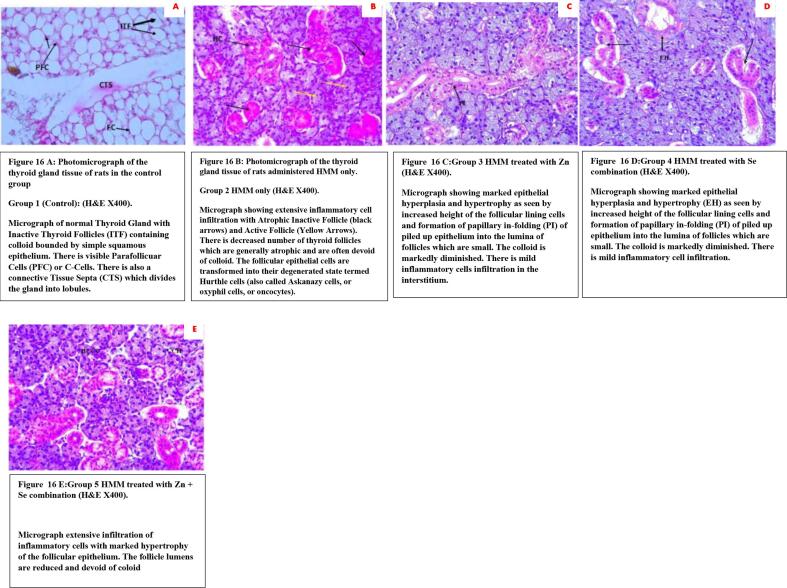
A: Photomicrograph of the thyroid gland tissue of rats in the control group. Group 1 (Control): (H&E X400). Micrograph of normal Thyroid Gland with Inactive Thyroid Follicles (ITF) containing colloid bounded by simple squamous epithelium. There is visible Parafollicuar Cells (PFC) or C-Cells. There is also a connective Tissue Septa (CTS) which divides the gland into lobules. Fig. 16 B: Photomicrograph of the thyroid gland tissue of rats administered HMM only. Group 2 HMM only (H&E X400). Micrograph showing extensive inflammatory cell infiltration with Atrophic Inactive Follicle (black arrows) and Active Follicle (Yellow Arrows). There is decreased number of thyroid follicles which are generally atrophic and are often devoid of colloid. The follicular epithelial cells are transformed into their degenerated state termed Hurthle cells (also called Askanazy cells, or oxyphil cells, or oncocytes). Fig. 16 C:Group 3 HMM treated with Zn (H&E X400). Micrograph showing marked epithelial hyperplasia and hypertrophy as seen by increased height of the follicular lining cells and formation of papillary in-folding (PI) of piled up epithelium into the lumina of follicles which are small. The colloid is markedly diminished. There is mild inflammatory cells infiltration in the interstitium. Fig. 16 D:Group 4 HMM treated with Se combination (H&E X400). Micrograph showing marked epithelial hyperplasia and hypertrophy (EH) as seen by increased height of the follicular lining cells and formation of papillary in-folding (PI) of piled up epithelium into the lumina of follicles which are small. The colloid is markedly diminished. There is mild inflammatory cell infiltration. Fig. 16 E:Group 5 HMM treated with Zn + Se combination (H&E X400).

The histology of representative rats from each group used in this study are demonstrated in Figs 16 A– E. The Photomicrograph of the thyroid gland tissue of a rat in the control group showed normal thyroid follicles and normal follicular cells (FC), Follicular cells colloid (FCC), Connective tissues (CT) and Para follicular cells (PFC) (Fig 16A), while the toxicity control (HMM alone) showed increased cellularity (IC) of thyroid follicles with monomorphic cells forming small follicular structures that are filled with colloid (Fig 16B). Zn treated thyroid of the HMM exposed female rats showed marked epithelial hyperplasia and hypertrophy (EH) as seen by increased height of the follicular lining cells and formation of papillary in-folding (PI) of piled up epithelium into the lumina of follicles which were small (Fig 16C). The colloid was markedly diminished. There was mild inflammatory cell infiltration. Zn + Se treated thyroid of the HMM exposed female rats showed extensive infiltration of inflammatory cells with marked hypertrophy of the follicular epithelium, with reduced follicle lumens devoid of colloid (Fig. 16 E).

### Discussion

This study has evaluated the protective effects of zinc and selenium in HMM mediated ovarian and thyroidal toxicity in animal model and demonstrated an appreciable attenuation of heavy metal-induced structural damage and dysfunction of the ovary and thyroid, oxidative stress, proinflammation and apoptosis, suggestive of ameliorative and beneficial effects of zinc and selenium. Co-existence of diverse environmental contaminants in food chains and water is a serious reason for worry because of the glued implication on public health and the environment. Significant pieces of evidence imply that concurrent exposure to different pollutants can trigger the toxicity of individual components (Lee et al., 2011). This study has demonstrated that exposure to HMM provoked oxido-inflammatory processes in the ovary and thyroid gland in female Wistar rats (Jovanović et al., 2013).

Thyroidal heavy metal sequestration has been demonstrated in this study. As envisaged, the ovary and thyroid gland accumulated substantial amount of the metals which led to severe histological distortions. Interestingly, significant reduction of the accumulated Pb, Al, Hg and Mn was observed in the ovary and thyroid of female rats that were co-treated with ETE. This reduction of ovary and thyroidal Pb, Al, Hg and Mn accumulation was remarkable and suggest that ETE could have enhanced the excretion of the absorbed metals evinced by high fecal matter Pb, Al, Hg and Mn levels (Table not shown). These observations are in consonance with the findings of Anyanwu et al., (2020). Se and Zn have been reported to reduce the absorption and bioaccumulation of toxic metals (Jemai et al., 2007, Xiao et al., 2002). Se and Zn supplementation in the present study decreased the levels of Pb, Hg, Al and Mn in the ovary and thyroid with evidence of higher levels of fecal elimination of these toxic metals and improved histoarchitecture which tended to agree with the findings of previous workers on the histoprotective effects of Se and Zn on heavy metal exposed thyroid gland of female rats (Salah et al 2022). Zn upregulates the activation of antioxidant transcription factors and the expression of genes for antioxidants and has been found to counteract oxidative stress in heavy metal-exposed rats (Samir et al., 2012, Adedara et al., 2019). Similarly, heavy metal mediated histopathological alterations which have been attenuated by Se and Zn administration were probably due to their antioxidant properties (Mohamed et al., 2016, Hassanin et al., 2013). HMM exposure caused significant changes in both the absolute and relative weight of the ovary whereas ETE supplementation tended to reverse these effects in the ovary.

NRF2 can be induced perniciously by ROS (Florczyk et al 2010) or non-perniciously (Kim et al., 2010, Yang et al., 2009). When there is exposure to oxidants or chemoprotective substances, the cysteine residues on the KEAP1-NRF2 complex senses cellular redox changes, resulting in structural alteration of KEAP1 and subsequent nuclear translocation of NRF2, heterodimerization with Maf and Jun bZip transcription factors, which bind to the 5¢-upstream *cis*-acting regulatory sequence known as the antioxidant response elements ARE (Hayes and McMahon 2001) and eventual induction transcription of Phase II antioxidant enzymes. In this study, Zn plus Se combination ETE supplementation boosted NRF2 expression in the ovary whereas Se boosted the NRF2 in the thyroid of HMM - exposed rats. This may suggest that the activation of NRF2 is one of the main protective mechanisms of ETE supplementation. Additionally, NRF2 may have exerted the cytoprotective effect by regulating NRF2 downstream target proteins expressions (namely, HMOX-1, SOD and CAT) when binding to ARE. Zn plus Se combination may have promoted nuclear transcription of NRF2 expressions as shown by higher levels of NRF2 in HMM co-administered Zn plus Se combination group than the control. This may suggest that Zn plus Se combination prevention of heavy metal -induced thyroid, toxicity may be linked to the activation of NRF2 signalling pathway. The higher level of HMOX-1 in the Zn treated group than the control and higher level of NRF2 in the Se treated group than the control may suggest beneficial antioxidant protective effect possibly via the NRF2-HMOX-1 signaling pathway in this study.

This study also investigated the effects of ETE on oxidative stress and apoptosis of HMM -exposed ovary and thyroid. Oxidative stress, marked by disproportion of pro-oxidation and the antioxidant defensive machinery, give rise to thyroid damage via amplification of lipid peroxidation and decreasing antioxidant enzyme activity (Luo et al., 2014). This study showed that ETE decreased the MDA (lipid peroxidation product and biomarker of oxidative stress) level but increased antioxidant enzyme activities (SOD and CAT) of thyroid of rats, exposed to HMM (Fu et al., 2013, Yu et al., 2019).

The increase in caspase-3 activity in the HMM-exposed rats which suggest the activation of the ovarian and thyroidal apoptotic pathways agrees with other studies that have implicated the involvement of caspase 3 dependent cascades in the heavy metal-induced apoptosis in different tissues (Kim and Sharma, 2006, Zwolak, 2020). On the contrary, ETE tended to abrogate the HMM-induced apoptosis.

Thyroid possesses an antioxidant enzymatic machinery which hinder generation of free radicals and their attendant deleterious effect. The first line of enzymatic antioxidant defense system (super oxide dismutase SOD) responsible for the catalytic conversion of superoxide radicals to less innocuous molecule H_2_O_2_, which is eventually converted to water by catalase CAT (Uner et al 2001). Exhaustion or saturation of these mechanisms activates the second line of antioxidant enzyme defense, predominantly mediated by glutathione peroxidase GPx (Duntas. 2012). Glutathione peroxidase GPx are cytoprotective against oxidative stress and lipid peroxidation, detoxification of H_2_O_2_ and lipid peroxides into less reactive species, using reduced glutathione GSH as a substrate (Sankar et al 2012). In this study, the ETE (Zn, Se and Zn + Se) had significant increase in CAT in comparison to the HMM only exposed rats.

In mammals, the overexpression of nitric oxide (NO) following exposure to any xenobiotic has been reported to damage the antioxidant system. Elevated levels of ROS and NO have been established to trigger diverse inflammatory response (Oboh et al., 2020, Seif et al., 2019). ETE markedly decreased the elevated NO levels in the ovary and thyroid, supportive of cytoprotective and antioxidant mechanism. In the present study HMM mediated upregulation of pro-inflammatory cytokines IL-6 and TNF-α in the ovary and thyroid were significantly decreased by ETE treatment. According to Diez et al., (2002), TNF-α plays a vital role in the expression of other cytokines involved in thyroid disorders, so it could have also triggered the over expression of IL – 6 and other cytokines in HMM treated rats as have been reported by previous researchers (Khayal et al., 2021).

Inflammatory response is usually amplified by oxidative stress through the modulation of associated genes (Cao et al., 2017, Tan et al., 2018). Oxidative stress provokes the activation of numerous transducers and redox sensitive transcription factor. The amplification of ovarian and thyroidal nuclear transcription factor (NF-kB) following exposure to HMM is an evidence of oxidative stress mediated inflammation which has been antagonized by ETE (Piazza et al., 2019).

Although some studies have suggested that the cytoprotective effect of Zn in heavy metal mediated toxicity may be due to redistribution of the heavy metals since Zn can elicit metallothionein synthesis, other studies have also shown that the antioxidant anti-apoptotic and anti-necrotic activities of Zn are also beneficial in the prevention of heavy metal mediated toxicity (Ueda et al., 1987, Jacquillet et al., 2006, Jemai et al., 2007, Imed et al., 2008). According to Imed and co-workers co-administration of Se and Zn following cadmium exposure did not significantly decrease the tissue levels of cadmium in comparison with the Cd–Zn group but there was an improved protection against heavy metal mediated histopathological damage in the Se–Zn group (Imed et al 2008). Available evidence tends to support that Zn and Se co-supplementation is better than that of Se or Zn. However, the assertion that Se supplementation may improve the protective effect of Zn in heavy metal-induced tissue damage maycall for further evaluation.

Supplementation with Se (El-Sharaky et al., 2007, Jamba et al., 2000, Newairy et al., 2007) or Zn (Jacquillet et al., 2006, Jamba et al., 2000) during heavy metal exposure have been shown to either prevent or diminish the noxious effects of the heavy metal on the cellular antioxidant machinery. In another study Imed and others demonstrated that the treatment of Cd-exposed rats with Se alone significantly increased the CAT activity, reversed Cd-induced increase in SOD activity and partially prevented Cd-induced decrease in GSH-Px activity, but did not reverse Cd-induced increase in MDA levels (Imed et al., 2009). The same study showed that supplementation with Zn alone, caused a significant increase in CAT activity and a partial protection against Cd-induced increase in the MDA concentrations (Imed et al 2009).

Hypofunctional endocrine status and low blood hormone levels associated with increasing body burden of aluminum related oxidative stress has been reported (Darbre, 2018). Ovarian failure arising from inadequate androgen hormone levels (Al-Eisa and Al-Nahari, 2017) and decreased androgen receptor function following aluminum exposure have also been documented (Gomes et al., 2019). Chronic metal exposure and accumulation affect many endocrine glands (Doumouchtsis et al 2009), namely affects the hypothalamic–pituitary axis, to give rise to blunted follicle-stimulating hormone (FSH)/luteinizing hormone (LH) responses to thyrotropin-releasing hormone, growth hormone–releasing hormone and gonadotropin-releasing hormone stimulation. Ovarian metal accumulation and damage to folliculogenesis seen in exposed animals manifest as decreased primordial follicles, increase of atretic antral follicle, edemas and necrosis of the ovarian follicles (Dhir and Dhand 2010., Krieg and Feng, 2011**.** Waseem, et al 2014). Mercury inhibits the release of luteinizing hormone (LH) and follicle-stimulating hormone (FSH) from the anterior pituitary (Massányi, et al 2020) and alter the levels of estrogen and progesterone. Mercury accumulation in the ovary of rats precipitates histo-morphometric distortions which include reduction of the total number of primordial, primary, and Graaf follicles, decrease in average mean volume of ovary, medulla and cortex, corpus luteum and Graaf follicles and a significant increase in the total volume of the atretic follicles (Altunkaynak et al 2016). The blunted follicle-stimulating hormone (FSH)/luteinizing hormone (LH) responses to thyrotropin-releasing hormone, growth hormone–releasing hormone and gonadotropin-releasing hormone stimulation can tip progesterone level as observed in this study which agree with previous studies (Ma et al 2018) were reversed by the ETE. These heavy metals bring about reduction in gonadotropin hormones and decreases in FSH and LH with extension in diestrus and shortening in proestrus but without significant elongation of cycle length (Massány et al 2020). One likely limitation of this study is the non-measurement of the thyroid will be addressed in future study.

The photomicrograph from the histological examination revealed degenerated ovarian cortex with follicles and theca cells in the ovary of the HMM treated groups when compared with the control group that showed normal ovarian cortex with primordial follicles. These observed lesions underscore the role of inflammation and oxidative stress in the progression of ovarian toxicity triggered by incessant HMM exposure. These histopathological findings validate the downregulation of female reproductive hormone observed in this study. Treatment with ETEs in this study improved the histologic lesions in the ovaries marked by recovered secondary follicles, mature ova, primordial and growing follicles, indicating recovery. These suggests possible antioxidant and anti-inflammatory properties of ETE. Heavy metals are associated with reduction in relative volume of primary follicles (Massanyi et al 1999), increased number of atretic primary follicles and atretic growing follicles (Nad’ et al., 2007) and significantly higher ovarian weight in comparison to controls (Massanyi et al 1999). Other studies have demonstrated dysfunction of folliculogenesis, marked by reduced primordial follicles and elevated atretic antral follicles, degeneration of the corpus luteum and fewer oocytes in the ovaries of the metal-exposed rats (Nasiadek et al 2019). The observed decrease in the number of follicles, with a distorted Graafian follicle in the ovaries of the HMM exposed female rats in this study is also in agreement with previous studies (Massány et al 2020). Ovarian heavy metal (Pb, Hg, Mn and Al) levels in this study were significantly higher in tissues/groups that showed more histopathological alterations which is in agreement with similar studies which reported higher Pb, Hg contents in damaged tissues than controls (Bjørklund et al., 2019, Al-Saleh et al., 2009, Merlo et al., 2019, Canaz et al., 2017).The interference of heavy metals with the development of growing follicles (Dhir, and Dhand 2010) evinced by atretic ovaries in all the stages suggestive of poor fertility were annulled by the ETE in this study.

The rats in the control group showed normal histological configuration in the thyroid section. The follicles at the boundary of the gland had few small peripheral vacuoles and were bigger than the ones at the central follicle with more colloids (Martini et al., 2012). The small and centrally dispersed follicles are responsible for the secretion of thyroid hormone while the follicles at the periphery predominantly serve as a pool of old hormones (Sosi-Jurjevi et al., 2006). In consonance with earlier reports on thyroid histology, the thyroid sections of the rats in HMM treated group showed histological alterations in comparison to the control group. The observed increase in the cellularity of thyroid follicles with monomorphic cells forming small follicular structures that are filled with colloid were in agreement with previous works on metals induced hypothyroidism Aboul-Fotouh *et al.* (2018). In the lumen, numerous follicles become degenerated with exfoliated epithelial cells, coalesce, collapse with distorted walls. Also, dark pyknotic nuclei and the cytoplasmic vacuolations observed in the follicular cells were in affirmation with those reported by Aboul-Fotouh *et al.* (2018).

Taken together, heavy metals can accumulate in tissues, to elicit histoarchitectural distortions, alter cytokine expression with upregulation of the pro-inflammatory markers (Zhu et al., 2012, Yu et al., 2019).

### Conclusion

In conclusion, ETE supplementation may provide appreciable protective effect against HMM-induced toxicity by inhibiting oxidative stress and apoptosis associated with activation of the NRF2 signalling pathway. Activation of the NRF2/ARE pathway by ETE supplementation may impair HMM -induced ovarian and thyroidal damage in female rats.

## CRediT authorship contribution statement

**Orish E. Orisakwe:** Conceptualization, Methodology, Project administration.

## Declaration of Competing Interest

The authors declare that they have no known competing financial interests or personal relationships that could have appeared to influence the work reported in this paper.

## Data Availability

No data was used for the research described in the article.
